# Prognostic value of GLIM-defined malnutrition in combination with hand-grip strength or gait speed for the prediction of postoperative outcomes in gastric cancer patients with cachexia

**DOI:** 10.1186/s12885-024-11880-z

**Published:** 2024-02-23

**Authors:** Zong-Ze Li, Xia-Lin Yan, Zhao Zhang, Jiong-Lai Chen, Jiang-Yuan Li, Jing-Xia Bao, Jia-Tong Ru, Jia-Xin Wang, Xiao-Lei Chen, Xian Shen, Dong-Dong Huang

**Affiliations:** 1 Department of Gastrointestinal Surgery, The First Affiliated Hospital of Wenzhou Medical University, 2 Fuxue Lane, 325000 Wenzhou, Zhejiang China; 2 Radiology Imaging Center, The First Affiliated Hospital of Wenzhou Medical University, Wenzhou, China

**Keywords:** Malnutrition, GLIM criteria, Gastric cancer, cachexia, Prognosis

## Abstract

**Background:**

Cancer cachexia is associated with impaired functional and nutritional status and worse clinical outcomes. Global Leadership Initiative in Malnutrition (GLIM) consensus recommended the application of GLIM criteria to diagnose malnutrition in patients with cachexia. However, few previous study has applied the GLIM criteria in patients with cancer cachexia.

**Methods:**

From July 2014 to May 2019, patients who were diagnosed with cancer cachexia and underwent radical gastrectomy for gastric cancer were included in this study. Malnutrition was diagnosed using the GLIM criteria. Skeletal muscle index was measured using abdominal computed tomography (CT) images at the third lumbar vertebra (L3) level. Hand-grip strength and 6-meters gait speed were measured before surgery.

**Results:**

A total of 356 patients with cancer cachexia were included in the present study, in which 269 (75.56%) were identified as having malnutrition based on the GLIM criteria. GLIM-defined malnutrition alone did not show significant association with short-term postoperative outcomes, including complications, costs or length of postoperative hospital stays. The combination of low hand-grip strength or low gait speed with GLIM-defined malnutrition led to a significant predictive value for these outcomes. Moreover, low hand-grip strength plus GLIM-defined malnutrition was independently associated with postoperative complications (OR 1.912, 95% CI 1.151–3.178, *P* = 0.012). GLIM-defined malnutrition was an independent predictive factor for worse OS (HR 2.310, 95% CI 1.421–3.754, *P* = 0.001) and DFS (HR 1.815, 95% CI 1.186–2.779, *P* = 0.006) after surgery. The addition of low hand-grip strength or low gait speed to GLIM-defined malnutrition did not increase its predictive value for survival.

**Conclusion:**

GLIM-defined malnutrition predicted worse long-term survival in gastric cancer patients with cachexia. Gait speed and hand-grip strength added prognostic value to GLIM-defined malnutrition for the prediction of short-term postoperative outcomes, which could be incorporated into preoperative assessment protocols in patients with cancer cachexia.

**Supplementary Information:**

The online version contains supplementary material available at 10.1186/s12885-024-11880-z.

## Introduction

Cancer cachexia is a challenging multifactorial clinical syndrome characterized by involuntary loss of skeletal muscle mass [1, 2]. Negative protein and energy balance driven by reduced food intake and abnormal metabolism play a key role in the pathogenesis of this syndrome [2]. In addition, other complex factors including neurological and immune system disorders are also involved in the progression of cancer cachexia [3]. Cancer cachexia was associated with impaired physical function, reduced quality of life and worse survival [1, 4]. Currently, there is no effective treatment that can reverse the progression of cancer cachexia [4]. Nutritional support is the mainstay of therapy for this syndrome [1, 4–6]. Therefore, adequate nutritional assessment is significant for patients with cancer cachexia for the guidance of its nutritional intervention. However, there is no consensus on the optimal nutritional assessment tool for patients with cancer cachexia.

The Global Leadership Initiative in Malnutrition (GLIM) Criteria was published in 2018 aiming to standardizing the diagnosis of malnutrition [7]. Since its introduction, GLIM-defined malnutrition has been widely applied in cancer patients and has been well recognized to be associated with adverse clinical outcomes in patients with cancer [8, 9]. The GLIM consensus recommended the application of GLIM criteria to diagnose malnutrition in persons with cachexia so that the priority to undertake appropriate nutrition interventions may be recognized [7]. However, few previous study has applied the GLIM criteria in patients with cancer cachexia.

Functional assessments such as hang-grip strength was recommended by the GLIM consensus as supportive measures. Our previous study has demonstrated that addition of low hang-grip strength or gait speed added prognostic value to GLIM-defined malnutrition in overweight patients [10]. A previous study has revealed a high prevalence of GLIM-defined malnutrition in patients with cancer cachexia defined by the international cancer cachexia consensus [11]. We speculated that additional assessment of hang-grip strength and gait speed besides GLIM-defined malnutrition can further risk-stratify patients with cancer cachexia.

Therefore, the objective of this study was to apply the GLIM criteria to gastric cancer patients with cachexia, and to investigate the predictive value of GLIM criteria for postoperative outcomes. We also aimed to investigate whether the combination of hand-grip strength or gait speed add prognostic value to GLIM-defined malnutrition for the prediction of postoperative outcomes in gastric cancer patients with cachexia.

## Methods

### Patients

The present study included gastric cancer patients diagnosed with cachexia in the First Affiliated Hospital of Wenzhou Medical University between July 2014 and May 2019. The international consensus defined cancer cachexia when one or more of the following three criteria was met: (a) weight loss > 5% over the past six months (in the absence of simple starvation); (b) BMI < 20 and any degree of weight loss > 2%; (c) low skeletal muscle index and any degree of weight loss > 2% [2]. Patients who received radical gastrectomy for gastric cancer with curative intent were included. The included individuals should have abdominal computed tomography (CT) images available for review within one month before surgery. Exclusions included patients who underwent palliative surgery or were unable to obtain muscle strength or physical function measurements. Patients were informed that their clinical information would be used anonymously for research and provided written informed consent for participation in the study. Informed written consent was obtained in accordance with the Declaration of Helsinki. This study and other related experiments were approved by the Ethical Review Board of The First Affiliated Hospital of Wenzhou Medical University. The present study was part of a large-scale observational study project registered in the China Clinical Trial Registry (No. ChiCTR1800019717).

### Data collection

The following data were collected prospectively, including (a) preoperative patient demographic characteristics, including gender, age, body mass index (BMI), hemoglobin concentration, serum albumin concentration, Charlson Comorbidity Index, previous abdominal operation; (b) the details of the surgery, including the type of resection, reconstruction, laparoscopy-assisted surgery, and combined resection; (c) tumor features include tumor location, TMN stage and degree of differentiation; (d) postoperative outcomes include complications within 30 days after surgery, costs and length of postoperative hospital stay. Complications were classified using the Clavien-Dindo classification. Complications rated as grade II or above were analyzed, and complications rated as grade III or higher were considered severe [12].

### Measurements of hand-grip strength, gait speed and muscle mass

Patients were tested for hand-grip strength and 6-meter gait speed before surgery, as previously reported [13]. Both genders’ cutoff values for low gait speed were 1 m/s, whereas the cutoff value for low handgrip strength was 26 kg for males and 18 kg for females, respectively [14]. Abdominal CT images at the third lumbar vertebra (L3) level were analyzed to measure the muscle mass, using a specialized imaging system (GE ADW4.5). Different tissue types were distinguished by the Hounsfield unit (HU) in the CT images. Skeletal muscle index (SMI, cm^2^/m^2^) was calculated to assess muscle mass. Based on our previous study, the threshold for low SMI was set at < 34.9 cm^2^/m^2^ in females and < 40.8 cm^2^/m^2^ in males [15]. Sarcopenia was defined by the combination of low hand-grip strength and low SMI. Severe sarcopenia was defined by additional low gait speed plus low hand-grip strength and low SMI [14].

### Diagnosis of malnutrition using GLIM criteria

In accordance with the GLIM consensus criteria, malnutrition was diagnosed using a two-step approach [7]. The first step was to screen patients at risk of malnutrition using the Nutritional Risk Screening 2002 (NRS2002). The presence of at least one phenotypic and one etiological criterion substantiated the diagnosis of malnutrition in the second step. Since patients with cancer had already met one of the etiological criteria (burden of disease), patients who met one of the three phenotypic criteria (weight loss, low BMI, and reduced muscle mass) were identified as malnourished. The three phenotypic standards were detailed as followed: [1] loss of weight: nonvoluntary weight loss of more than 5% within the previous six months or more than 10% of any time; [2] low BMI: BMI of less than 20 kg/m^2^ for patients older than 70 years or less than 18.5 kg/m^2^ for those younger than 70 years; and [3] low muscle mass: assessed by SMI based on abdomen CT scans. As previously described, the cut-off values for low SMI were referenced from our previous study [15].

### Follow-up

Patients were followed up one month after surgery, every three months for the first two years, and then every six months after that. The main follow-up components include medical history, physical examination, laboratory tests, imagological examinations, and endoscopy if necessary. Moreover, patients were contacted by regular telephone calls to assist with the follow-up program. Overall survival (OS) was defined by the period from the date of radical gastrectomy for gastric cancer to the date of death from any cause. Disease-free survival (DFS) was calculated from the date of radical gastrectomy to the date of cancer recurrence or death from any cause, whichever occurred first.

### Statistical analysis

Data were presented as means with standard deviations (SD) or, when appropriate, as medians and interquartile ranges (IQR). The t-test or Mann-Whitney U test was used to compare continuous data, and the Chi-squared test or Fisher’s exact test was used to compare categorical data. Kaplan–Meier curves were plotted, and the log-rank test was applied to test the difference in survival. Univariate analysis was used to identify potential risk factors for OS and DFS. Multivariate Cox regression analysis was used to identify independent risk factors for survival. Two-tailed P values < 0.05 were considered statistically significant. The data were analyzed using SPSS statistics version 22.0 (IBM, USA).

## Results

### Baseline characteristics of patients

A total of 356 patients who were diagnosed with cancer cachexia were included in the present study. According to the GLIM criteria, 269 (75.56%) patients were defined as malnourished. Compared with non-malnourished patients, patients with GLIM-defined malnutrition were older, had a lower BMI and SMI, had lower preoperative albumin and hemoglobin levels, had a lower 6-meter gait speed, and had a higher incidence of previous abdominal surgery (Table 1).

**Table 1:** Baseline characteristics of patients

Characteristics	Total (n = 356)	GLIM-defined malnutrition (n = 269)	Non-malnutrition (n = 87)	P
Age, median (IQR), years	66 (14)	68 (15.5)	62 (11)	< 0.001^*^
Gender				0.062
Female	122 (34.3%)	85 (31.6%)	37 (42.5%)	
Male	234 (65.7%)	184 (68.4%)	50 (57.5%)	
BMI, median (IQR), kg/m^2^	20.90 (2.22)	20.45 (2.21)	22.11 (1.98)	< 0.001^*^
Albumin, mean (SD), g/L	36.93 ± 4.97	36.42 ± 4.87	38.30 ± 4.96	< 0.001^*^
Hemoglobin, median (IQR), g/L	119.00 (15.75)	116.00 (16.00)	127.00 (13.00)	0.006^*^
Charlson Comorbidity Index				0.343
0	250 (70.2%)	185 (68.8%)	65 (74.7%)	
1	68 (19.1%)	55 (20.4%)	13 (14.9%)	
≥ 2	38 (10.7%)	29 (10.8%)	9 (10.4%)	
Previous abdominal surgery				0.045^*^
Yes	47 (13.2%)	30 (11.2%)	17 (19.5%)	
No	309 (86.8%)	239 (88.8%)	70 (80.5%)	
Weight loss in the last 6 months (IQR), (%)	7.38 (4.69)	7.85 (4.28)	4.08 (3.81)	< 0.001*
L3 SMI, median (IQR), cm^2^/m^2^	39.66 (4.51)	39.57 (4.28)	40.23 (6.14)	0.081
SMD, median (IQR), HU	36.70 (4.30)	36.20 (4.85)	37.50 (5.50)	0.334
HGS, mean (SD), kg	24.90 ± 8.91	24.44 ± 8.94	26.34 ± 8.73	0.084
6-m gait speed, mean (SD), m/s	0.96 ± 0.23	0.94 ± 0.23	1.02 ± 0.22	0.004^*^
Tumor location				0.996
Proximal	48 (13.5%)	36 (13.4%)	12 (13.8%)	
Medium	75 (21.1%)	56 (20.8%)	19 (21.8%)	
Distal	218 (61.2%)	165 (61.3%)	53 (60.9%)	
2/3 or more	15 (4.2%)	12 (4.5%)	3 (3.5%)	
Differentiation of tumor				0.563
Poorly differentiated	249 (69.9%)	186 (69.1%)	63 (72.4%)	
Well differentiated	107 (30.1%)	83 (30.9%)	24 (27.6%)	
TNM stage				0.690
I	88 (24.7%)	61 (22.7%)	27 (31.0%)	
II	85 (23.9%)	71 (26.4%)	14 (16.1%)	
III	183 (51.4%)	137 (50.9%)	46 (52.9%)	
Laparoscopy assisted surgery				0.371
Yes	117 (32.9%)	85 (31.6%)	32 (36.8%)	
No	239 (67.1%)	184 (68.4%)	55 (63.2%)	
Type of resection				0.128
Subtotal gastrectomy	201 (56.5%)	158 (58.7%)	43 (49.4%)	
Total gastrectomy	155 (43.5%)	111 (41.3%)	44 (50.6%)	
Combined organ resection				0.554
Yes	30 (8.4%)	24 (8.9%)	6 (6.9%)	
No	326 (91.6%)	245 (91.1%)	81 (93.1%)	

IQR, interquartile range; SD, standard deviation; BMI, body mass index; L3, third lumbar vertebra; SMI, skeletal muscle index; SMD, skeletal muscle density; HU, Hounsfield unit; HGS, hand-grip strength; TNM, tumor–node–metastasis.

The values in the table were number of patients and percent unless indicated otherwise.

^*^ Statistically significant compared with patients without malnutrition.

### Shor-Term postoperative outcomes

Postoperative complications occurred in 102 (28.7%) of the patients with cancer cachexia. There was no significant difference between the patients with and without GLIM-defined malnutrition in the short-term postoperative outcomes, including postoperative complications, costs, and postoperative hospital stays (Table 2). A total of 115 individuals (42.8%) exhibited low hand-grip strength, and 85 (31.6%) had low gait speed in addition to having GLIM-defined malnutrition. Notably, the addition of low hand-grip strength or low gait speed to GLIM-defined malnutrition led to a significant predictive value for a higher incidence of postoperative complications, higher costs, and longer postoperative hospital stays. Moreover, low gait speed plus GLIM-defined malnutrition predicted severe postoperative complications (Table 2). Table 3 provides a list of the sensitivity and specificity of several malnutrition diagnoses for predicting postoperative complications. Multivariate analysis showed that GLIM-defined malnutrition plus low hand-grip strength, low albumin, and combined organ resection were independent risk factors for postoperative complications. In contrast, laparoscopic surgery was an independent protective factor (Table 4).

**Table 2:** Details of short-term postoperative outcomes

Complications		Total (n = 356)	GLIM-defined malnutrition (n = 269)	GLIM-defined malnutrition + low gait speed (n = 85)	GLIM-defined malnutrition + low hand-grip strength (n = 115)
**Total complications** ^†^		102 (28.7%)	81 (31.6%)	34 (40.0%)^*^	47 (40.9%)^*^
	Hydrothorax	9 (2.5%)	6 (2.2%)	4 (4.7%)	4 (3.5%)
	Incision infection	11 (3.1%)	9 (3.3%)	2 (2.4%)	4 (3.5%)
	Abdominal hemorrhage	9 (2.5%)	8 (3.0%)	5 (5.9%)	4 (3.5%)
	Abdominal infection	19 (5.3%)	12 (4.5%)	3 (3.5%)	4 (3.5%)
	Peritoneal fluid accumulation	12 (3.4%)	9 (3.3%)	2 (2.4%)	6 (5.2%)
	Pneumonia	18 (5.1%)	15 (5.6%)	9 (10.6%)^*^	14 (12.2%)^*^
	Deep vein thrombosis	3 (0.8%)	2 (0.7%)	1 (1.2%)	1 (0.9%)
	Pulmonary embolism	3 (0.8%)	2 (0.7%)	1 (1.2%)	2 (1.7%)
	Intestinal obstruction	6 (1.7%)	3 (1.1%)	2 (2.4%)	2 (1.7%)
	Decreased gastrointestinal motility	11 (3.1%)	10 (3.7%)	3 (3.5%)	5 (4.3%)
	Anastomotic fistula	5 (1.4%)	3 (1.1%)	2 (2.4%)	0 (0%)
	Stump fistula	5 (1.4%)	5 (1.9%)	2 (2.4%)	4 (3.5%)
	Pancreatic or biliary fistula	5 (1.4%)	5 (1.9%)	1 (1.2%)	2 (1.7%)
	Septic shock	2 (0.6%)	1 (0.4%)	1 (1.2%)	1 (0.9%)
	Other	8 (2.2%)	8 (3.0%)	5 (5.9%)^*^	5 (4.3%)
**Severe complications** ^‡^		23 (6.5%)	19 (7.0%)	10 (11.8%)^*^	11 (9.6%)
**Length of postoperative stays, median (IQR), days**		13 (7)	13 (7)	14 (9.5)^*^	14 (9)^*^
**Costs, median (IQR), RMB**		63025.56 (25038.59)	63617.50 (25981.47)	72189.68 (20140.80)^*^	69732.21 (28041.21)^*^

IQR, interquartile range

The values in the table were number of patients and percent unless indicated otherwise.

^†^ Complications classified as grade II and above.

^‡^ Complications classified as grade III and above.

^*^ Statistically significant compared with the opposite group.

**Table 3:** Sensitivity and specificity of different malnutrition diagnoses for the prediction of postoperative complications^†^

Factors	Sensitivity	Specificity	Accuracy	PPV	NPV
GLIM-defined malnutrition	79.41%	25.98%	41.29%	30.11%	75.86%
GLIM-defined malnutrition + low gait speed	33.33%	79.92%	66.57%	40.00%	74.91%
GLIM-defined malnutrition + low hand-grip strength	46.08%	73.23%	65.45%	40.87%	77.18%

PPV, positive predictive value; NPV, negative predictive value.

^†^ Postoperative complications classified as Grade II or above by the Clavien-Dindo classification were analyzed.

**Table 4:** Univariate and multivariate logistic regression analysis of risk factors for postoperative complications

Factors	Univariable analysis		Multivariate analysis	
	OR (95%CI)	P	OR (95%CI)	P
GLIM-malnutrition				
Yes/No	1.354 (0.777–2.361)	0.285		
GLIM-malnutrition + low HGS				
Yes/No	2.337 (1.449–3.771)	< 0.001^*^	1.912 (1.151–3.178)	0.012^*^
GLIM-malnutrition + low gait speed				
Yes/No	1.990 (1.191–3.326)	0.009^*^		
Age, years				
≥ 75/<75	2.307 (1.354–3.934)	0.002^*^		
Gender				
Males/females	1.060 (0.652–1.724)	0.814		
BMI, kg/m2				
< 18.5/≥18.5	1.433 (0.838–2.450)	0.189		
Low albumin				
Yes/No	2.753 (1.698–4.461)	< 0.001^*^	2.086 (1.251–3.480)	0.005^*^
Anemia				
Yes/No	2.184 (1.369–3.485)	0.001^*^		
Charlson Comorbidity Index		0.031^*^		
1/0	1.004 (0.547–1.843)	0.991		
≥ 2/0	2.509 (1.251–5.033)	0.010^*^		
Previous abdominal surgery				
Yes/No	0.945 (0.476–1.875)	0.872		
Tumor location		0.798		
Medium/ Proximal	1.245 (0.548–2.830)	0.600		
Distal/ Proximal	1.275 (0.624–2.605)	0.506		
2/3 or more / Proximal	0.750 (0.181–3.115)	0.692		
Differentiation of tumor				
Poorly /Well	1.468 (0.871–2.475)	0.149		
TNM stage		0.462		
II/I	1.255 (0.636–2.479)	0.513		
III/I	1.443 (0.807–2.582)	0.216		
Laparoscopy-assisted surgery				
Yes/No	0.496 (0.292–0.842)	0.009^*^	0.553 (0.319–0.959)	0.035^*^
Type of resection				
Total/Subtotal	1.221 (0.770–1.938)	0.396		
Combined organ resection				
Yes/No	2.747 (1.289–5.854)	0.009^*^	2.222 (1.007–4.905)	0.048^*^

OR, odds ratio; CI, Confidence interval; BMI, body mass index; SMI, skeletal muscle index; HGS, hand-grip strength; TNM, tumor–node–metastasis.

^*^ Statistically significant.

### Long-term prognosis

**Fig. 1:**
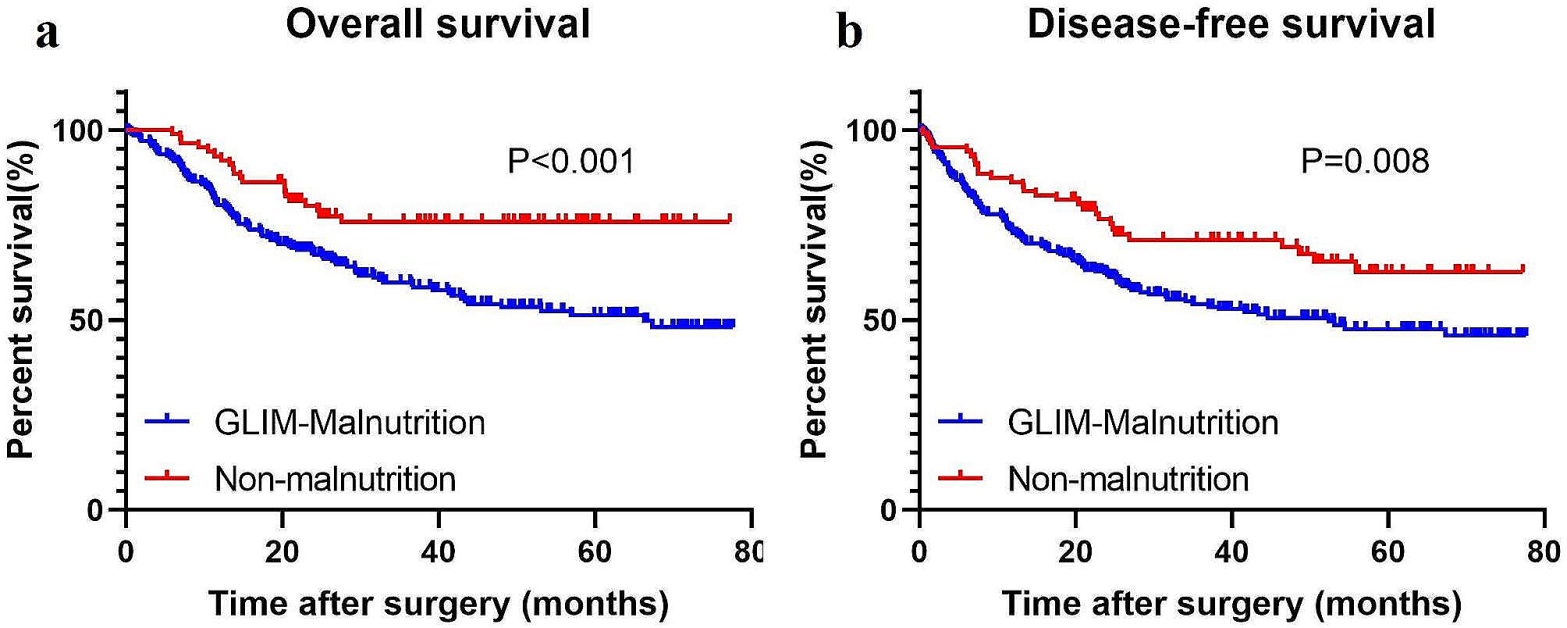
Survival curves of overall survival (**a**) and disease-free survival (**b**) in patients with and without malnutrition based on the GLIM criteria

The median follow-up time was 46.6 months. GLIM-defined malnutrition was associated with worse OS and DFS (Fig. 1). Multivariate Cox regression analysis showed that GLIM-defined malnutrition, age ≥ 75 years, poorly differentiated tumor, high TMN stage, total gastrectomy and combined organ resection were independent risk factors for OS (Table 5), whereas GLIM-defined malnutrition, BMI < 18.5, poorly differentiated tumor, high TMN stage, total gastrectomy and combined organ resection were independent risk factors for DFS (Table 6). The addition of low hand-grip strength or low gait speed to GLIM-defined malnutrition did not increase its predictive value for OS or DFS (Tables 5 and 6).

**Table 5:** Univariate and multivariate Cox regression analysis of factors associated with overall survival

Factors	Univariable analysis		Multivariate analysis	
	HR (95%CI)	P	HR (95%CI)	P
GLIM-malnutrition				
Yes/No	2.185 (1.356–3.519)	0.001^*^	1.892 (1.165–3.075)	0.010^*^
GLIM-malnutrition + low HGS				
Yes/No	1.288 (0.897–1.850)	0.171		
GLIM-malnutrition + low gait speed				
Yes/No	1.871 (1.289–2.716)	0.001^*^		
Age, years				
≥ 75/<75	1.494 (1.015–2.201)	0.042^*^	1.582 (1.065–2.350)	0.023^*^
Gender				
Males/females	0.990 (0.691–1.417)	0.955		
BMI, kg/m2				
< 18.5/≥18.5	1.571 (1.001–2.466)	0.049^*^		
Low albumin				
Yes/No	1.520 (1.066–2.166)	0.021^*^		
Anemia				
Yes/No	1.528 (1.085–2.154)	0.015^*^		
Charlson Comorbidity Index		0.472		
1/0	0.746 (0.465–1.197)	0.225		
≥ 2/0	0.996 (0.568–1.747)	0.990		
Previous abdominal surgery				
Yes/No	0.510 (0.268–0.972)	0.041^*^		
Tumor location		0.104		
Medium/ Proximal	1.067 (0.581–1.958)	0.834		
Distal/ Proximal	1.028 (0.608–1.739)	0.917		
2/3 or more / Proximal	2.330 (1.067–5.092)	0.034^*^		
Differentiation of tumor				
Poorly /Well	2.048 (1.534–3.779)	< 0.001^*^	1.692 (1.063–2.693)	0.027^*^
TNM stage		< 0.001^*^		< 0.001^*^
II/I	1.716 (0.816–3.606)	0.154	1.222 (0.574–2.603)	0.603
III/I	5.719 (3.067–10.663)	< 0.001^*^	4.180 (2.192–7.971)	< 0.001^*^
Laparoscopy-assisted surgery				
Yes/No	0.550 (0.362–0.837)	0.005^*^		
Type of resection				
Total/Subtotal	1.954 (1.381–2.764)	< 0.001^*^	1.483 (1.035–2.125)	0.032^*^
Combined organ resection				
Yes/No	2.700 (1.675–4.352)	< 0.001^*^	1.892 (1.165–3.075)	0.010^*^

HR, Hazard ratio; CI, Confidence interval; BMI, body mass index; SMI, skeletal muscle index; HGS, hand-grip strength; TNM, tumor–node–metastasis.

^*^ Statistically significant.

**Table 6:** The results of the univariate and multivariate Cox regression analysis for disease-free survival

Factors	Univariable analysis		Multivariate analysis	
	OR (95%CI)	P	OR (95%CI)	P
GLIM-malnutrition				
Yes/No	1.728 (1.145–2.607)	0.009^*^	1.815 (1.186–2.779)	0.006^*^
GLIM-malnutrition + low HGS				
Yes/No	1.216 (0.864–1.710)	0.261		
GLIM-malnutrition + low gait speed				
Yes/No	1.760 (1.237–2.503)	0.002^*^		
Age, years				
≥ 75/<75	1.263 (0.868–1.838)	0.222		
Gender				
Males/females	1.126 (0.800-1.584)	0.495		
BMI, kg/m2				
< 18.5/≥18.5	1.573 (1.098–2.253)	0.014^*^	1.567 (1.021–2.406)	0.040^*^
Low albumin				
Yes/No	1.635 (1.176–2.273)	0.003^*^		
Anemia				
Yes/No	1.563 (1.134–2.154)	0.006^*^		
Charlson Comorbidity Index				
1/0	0.749 (0.482–1.166)	0.201		
≥ 2/0	1.048 (0.628–1.748)	0.858		
Previous abdominal surgery				
Yes/No	0.752 (0.448–1.262)	0.281		
Tumor location		0.063		
Medium/ Proximal	1.298 (0.731–2.306)	0.373		
Distal/ Proximal	1.156 (0.696–1.920)	0.574		
2/3 or more / Proximal	2.590 (1.223–5.487)	0.013^*^		
Differentiation of tumor				
Poorly /Well	2.630 (1.711–4.044)	< 0.001^*^	1.838 (1.184–2.854)	0.007^*^
TNM stage		< 0.001^*^		< 0.001^*^
II/I	1.901 (0.968–3.735)	0.062	1.522 (0.769–3.010)	0.228
III/I	5.817 (3.273–10.337)	< 0.001^*^	4.259 (2.363–7.678)	< 0.001^*^
Laparoscopy-assisted surgery				
Yes/No	0.616 (0.423–0.898)	0.012^*^		
Type of resection				
Total/Subtotal	1.979 (1.432–2.736)	< 0.001^*^	1.513 (1.085–2.110)	0.015^*^
Combined organ resection				
Yes/No	2.840 (1.804–4.472)	< 0.001^*^	2.434 (1.531–3.870)	< 0.001^*^

HR Hazard ratio; CI Confidence interval; BMI, body mass index; SMI, skeletal muscle index; HGS, hand-grip strength; TNM, tumor–node–metastasis.

^*^ Statistically significant.

### Influence of malnutrition severity on the outcomes

Within the 269 malnutritional patients, 150 (55.8%) were categorized as moderate malnutrition and 119 (44.2%) were categorized as severe malnutrition. No significant difference was found between the two groups in term of postoperative complications, costs, length of postoperative stays (Supplementary Table 1) and long-term survivals (Supplementary Fig. 1).

### Influence of GLIM-defined malnutrition on cachexia patients categorized by severity of Sarcopenia

Cachectic patients were further categorized into ‘non-sarcopenia’, ‘sarcopenia, not severe’, and ‘severe sarcopenia’ sub-groups. The prognostic value of GLIM-defined malnutrition were analyzed in these sub-groups. No significant association was found between GLIM-defined malnutrition and short-term outcomes in these sub-groups (Supplementary Table 2). For the long-term prognosis, GLIM-defined malnutrition was associated with worse OS and DFS in cachexia patients without sarcopenia, but not in cachexia patients with sarcopenia or severe sarcopenia (Supplementary Fig. 2).

## Discussion

As far as we know, the present study was the first study to investigate the prognostic value of GLIM-defined malnutrition in patients with cancer cachexia. In the present study, we found a high prevalence (75.56%) of malnutrition as defined by the GLIM criteria among cachexia patients with resectable gastric cancer. Although GLIM-defined malnutrition alone did not show significant association with postoperative complications, costs or length of postoperative hospital stays, the combination of low hand-grip strength or low gait speed with GLIM-defined malnutrition led to a significant predictive value for these outcomes. GLIM-defined malnutrition was an independent predictive factor for worse OS and DFS after surgery. The addition of low hand-grip strength or low gait speed to GLIM-defined malnutrition did not increase its predictive value for survival.

Cancer cachexia is a complex metabolic syndrome caused by tumor-related factors, characterized by increased resting energy expenditure (REE) and impaired balance between skeletal muscle synthesis and breakdown [16]. Reduced food intake and increased catabolism induced by cancer cachexia both lead to the occurrence of malnutrition. In fact, there is a debate in the concepts of malnutrition and cachexia in the literature. In 2010, European Society for Clinical Nutrition and Metabolism (ESPEN) Special Interest Groups (SIG) agreed on considering malnutrition and cachexia as two different conditions, but they indicated that there is overlap between the two conditions [17]. In 2017, ESPEN released guidelines on the definitions and terminology of clinical nutrition, in which cachexia was considered a synonym for chronic disease-related malnutrition (DRM) with inflammation [18]. Similarly, a recent opinion paper by Muscaritoli et al. described cachexia as DRM with inflammation and emphasized the importance of evaluating inflammation in distinguishing between malnutrition associated with abnormal catabolism and malnutrition associated with insufficient nutrient intake and assimilation [19]. Based on this definition, it seems counterintuitive that 24.44% of patients with cachexia do not appear to be malnourished according to the GLIM criteria in our study. However, our analysis showed that patients with cancer cachexia can be further stratified into malnutritional and non-malnutritional defined by the GLIM criteria, and that GLIM-defined malnutrition had significant prognostic value for patients with cancer cachexia. It is noteworthy that current international consensus definition of cancer cachexia was published in 2011. Our results suggested that current definition of cancer cachexia could not adequately reflect the malnutritional condition of the patients, which indicated the limitations of the current definition of cancer cachexia. Another recent multicenter study showed that 11.2% patients with cancer cachexia defined by the international consensus was not malnourished according to the GLIM criteria [11], which also indicated the limitations and incomplete overlap of the GLIM-defined malnutrition and the 2011 international cancer cachexia consensus definitions. Therefore, the conclusion of the present study was based on current diagnostic criteria of cancer cachexia and GLIM criteria of malnutrition. Our present study provided new evidence for the improvement of the definition of malnutrition and cancer cachexia.

In our study, we found that GLIM-defined malnourished patients were older, had lower BMI and SMI, had lower levels of pre-operative albumin and hemoglobin, and a lower gait speed. BMI and SMI represent body composition, whereas gait speed reflects physical functional status. Albumin and hemoglobin levels are traditional biochemical indices associated with nutritional status. Therefore, our results demonstrated that the GLIM-defined malnutrition was a indicator for the nutritional and functional status in patients with cancer cachexia.

The present study identified GLIM-defined malnutrition as an independent predictor for OS and DFS in gastric cancer patients with cachexia. Previous studies have demonstrated that GLIM-defined malnutrition was associated with six-month and one-year survival in cancer patients [20, 21]. Cancer is a chronic wasting disease. Nutritional status reflects patient’s functional reserve in the confrontation with the malignant disease. Malnutrition is one of the direct causes of mortality in cancer patients [22]. Moreover, malnutrition can reduce the patients’ tolerance for oncological therapies, which can in turn exacerbate the tumor progression [23]. Cachexia is well known to be associated with worse prognosis in cancer patients [24]. Our finding demonstrated that identifying malnutrition can further risk-stratify the cachexia patients for the long-term survival. Although cancer cachexia cannot be reversed by conventional nutritional support [25, 26], the clinical outcomes of cachexia patients can be improved by nutritional interventions. A multicenter prospective cohort study proved that enteral nutrition and parenteral nutrition can improve survival in patients with advanced cancer cachexia [6]. Therefore, our results indicated that GLIM criteria can be used as a good nutritional assessment tool for the guidance of nutritional support in cancer cachexia patients.

The relationship between GLIM-defined malnutrition and postoperative complications has been reported by many previous studies [27–29]. In contrast with these previous studies, the present study did not find a significant association between GLIM-defined malnutrition and postoperative complications in patients with cancer cachexia. The lack of prognostic value of GLIM-related malnutrition may depend on the choice of study population. GLIM-defined malnourished cachectic patients are compared with non-malnourished but cachectic patients, which may explain the lack of statistical significance. However, GLIM-defined malnutrition plus low hand-grip strength or low gait speed predicted the occurrence of postoperative complications. Similarly, one of our previous study showed that GLIM-defined malnutrition alone was not predictive for postoperative complications, and the addition of low gait speed or muscle quality to GLIM-defined malnutrition led to a significant predictive value for postoperative complications in overweight patients with gastric cancer [10]. Our finding indicated that GLIM-defined malnutrition alone was not sufficient to predict postoperative complications in certain types of patients, such as overweight patients or patients with cancer cachexia. Measurement of muscle strength and physical functional have additional prognostic value besides GLIM-defined malnutrition and should be incorporated in the routine preoperative assessments in patients with cancer cachexia.

In the present study, BMI < 18.5 was associated with worse OS and DFS and was identified as an independent risk factor for DFS. For patients with cancer, BMI < 18.5 is one of the diagnostic criteria of malnutrition in many guidelines, including ESPEN [30] and GLIM [7]. However, patients with BMI < 18.5 constituted only 14.0% (50/356) in patients with cachexia. This proportion is much lower in the overall population of cancer patients. Therefore, BMI < 18.5 cannot be used as a sensitive index in nutritional screening and assessment but can be used as a strong indicator for poor nutritional status and worse outcomes in preoperative risk stratification.

Total gastrectomy and combined organ resection were identified as independent risk factors for worse OS and DFS after surgery in our study. Total gastrectomy and combined organ resection are relatively aggressive operations aiming to achieve more extensive oncological resection. Total gastrectomy is performed in patients with proximal gastric cancer, which provide more extensive lymph node resection compared with proximal gastrectomy [31]. However, total gastrectomy result in a poorer nutritional status after surgery compared with proximal gastrectomy [32, 33]. Malnutrition was significantly associated with the intolerance of oncological therapies and was an independent predictor for survival as revealed by the present study and other previous studies [20, 21]. This can partially explain the negative impact of total gastrectomy on survival in the present study. Previous study has demonstrated that combined organ resection resulted in better 5-year survival rates in patients with infiltrating gastric cancer invading adjacent organs without metastasis [34], which was in contrast with the finding of the present study. In the present study, the incidence of overall complications and severe complications were 50% and 20% in patients who received combined organ resection, which were much higher compared with that in patients who did not receive combined organ resection (22.1% and 5.2%, respectively). Moreover, combined organ resection was an independent risk factor for postoperative complications as revealed by the present study (Table 4). Postoperative complications can result in worse long-term prognosis [35, 36], which can partially explain the negative influence of combined organ resection on survival in the present study. Therefore, our results indicated that total gastrectomy and combined organ resection should be chosen cautiously in gastric cancer patients with cachexia.

## Conclusion

The present study showed that GLIM criteria are good nutritional assessment tools in patients with cancer cachexia. GLIM-defined malnutrition independently predicted worse long-term survival in gastric cancer patients with cachexia. Gait speed and hand-grip strength added prognostic value to GLIM-defined malnutrition for the prediction of short-term postoperative outcomes, which could be incorporated into preoperative assessment protocols in patients with cancer cachexia.

### Electronic supplementary material

Below is the link to the electronic supplementary material.

Supplementary Material 1

Supplementary Material 2

Supplementary Material 3

Supplementary Material 4

Supplementary Material 5

## Data Availability

The datasets generated during and/or analyzed during the current study are available from the corresponding author on reasonable request.

## Abbreviations

GLIM: Global Leadership Initiative in Malnutrition
L3: Third lumbar vertebra
CT: Computed tomography
OR: Odds ratio
HR: Hazard ratio
CI: Confidence interval
BMI: Body mass index
SMI: Skeletal muscle index
SMD: Skeletal muscle density
HU: Hounsfield unit
HGS: Hand-grip strength
TNM: Tumor–node–metastasis
DFS: Third lumbar vertebra
IQR: Interquartile range
SD: Standard deviation
DFS: Disease-free survival
OS: Overall survival
REE: Resting energy expenditure
