# Supplementary material for: Prognostic value of GLIM-defined malnutrition in combination with hand-grip strength or gait speed for the prediction of postoperative outcomes in gastric cancer patients with cachexia
Source: BMC Cancer. 2024 Feb 23;24:253. doi: 10.1186/s12885-024-11880-z (PMC10885679; doi:10.1186/s12885-024-11880-z)
Supplement: Supplementary file 1 — Supplementary Material 1 [file 12885_2024_11880_MOESM1_ESM.docx]

**Supplementary Table 1. Influence of malnutrition** **severity on the short-term outcomes**

| **Short-term outcomes** | GLIM-defined malnutrition, moderate (n =150) | GLIM-defined malnutrition, severe (n =119) | P |
| --- | --- | --- | --- |
| **Total complications**^†^ | 48 (32.0%) | 33 (27.7%) | 0.448 |
| **Severe complications**^‡^ | 9 (6.0%) | 10 (8.4%) | 0.445 |
| **Length of postoperative stays, median (IQR), days** | 13(7.25) | 14 (7) | 0.319 |
| **Costs, median (IQR), RMB** | 62664.67 (24597.24) | 68696.78 (27705.38) | 0.052 |

IQR, interquartile range

The values in the table were number of patients and percent unless indicated otherwise.

^†^ Complications classified as grade II and above.

^‡^ Complications classified as grade III and above.

^*^ Statistically significant compared with the opposite group.
