# Supplementary figures and images for: Prognostic value of GLIM-defined malnutrition in combination with hand-grip strength or gait speed for the prediction of postoperative outcomes in gastric cancer patients with cachexia
Source: BMC Cancer. 2024 Feb 23;24:253. doi: 10.1186/s12885-024-11880-z (PMC10885679; doi:10.1186/s12885-024-11880-z)

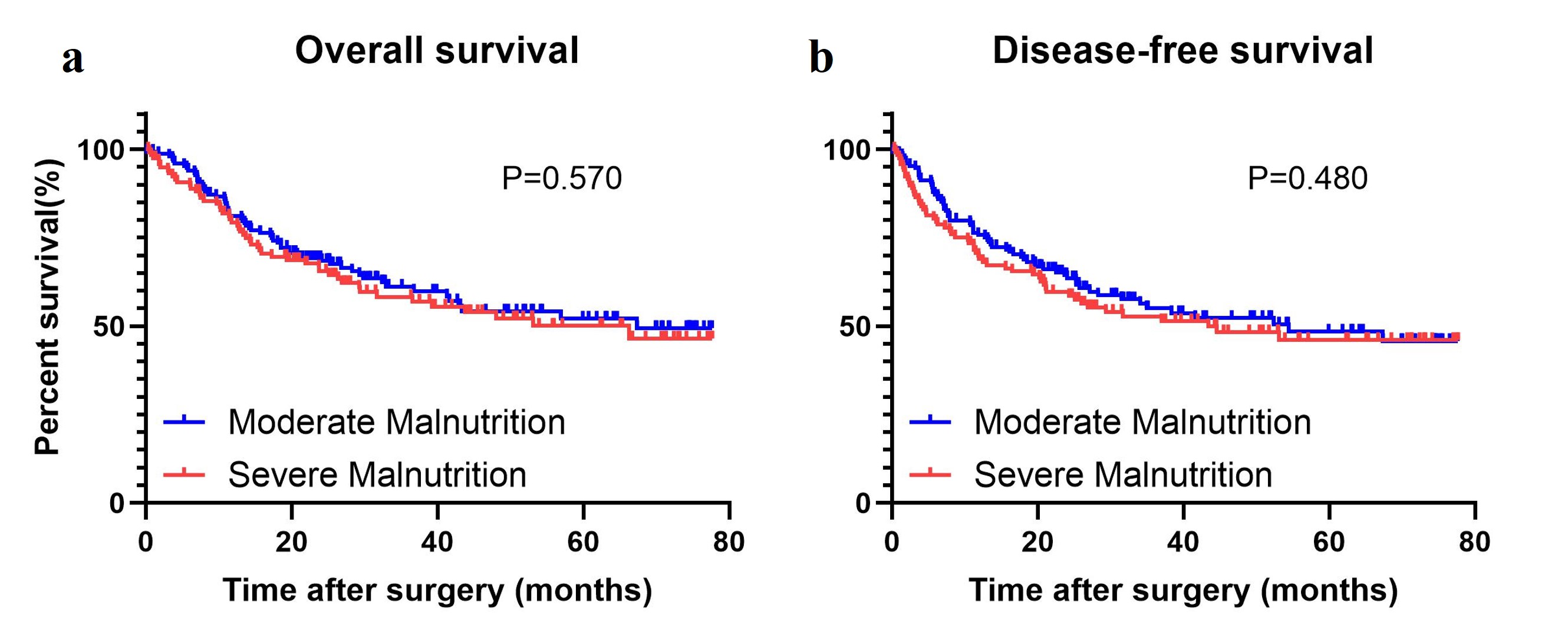

Supplement: Supplementary file 2 — Supplementary Material 2 [file 12885_2024_11880_MOESM2_ESM.jpg]

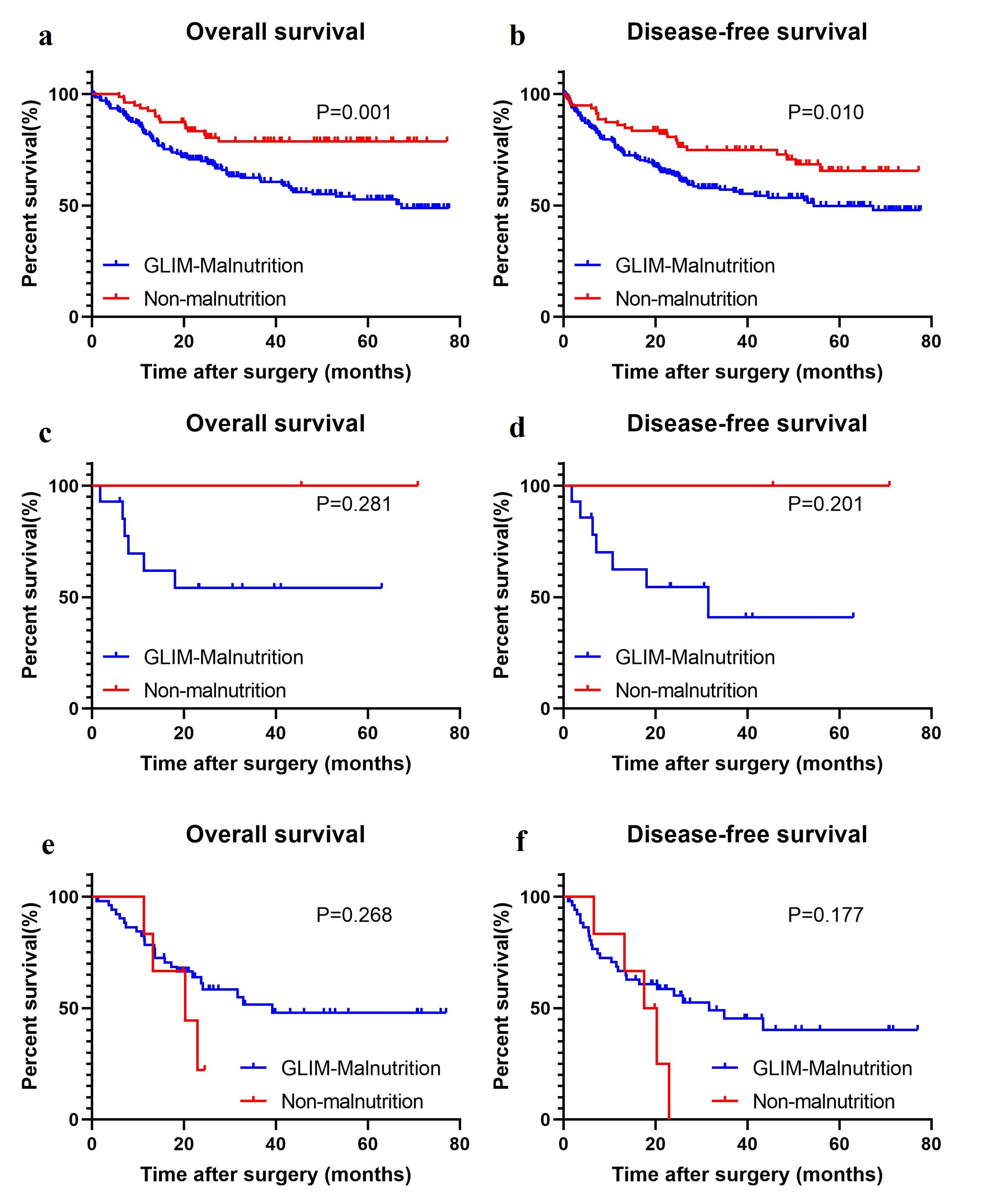

Supplement: Supplementary file 4 — Supplementary Material 4 [file 12885_2024_11880_MOESM4_ESM.jpg]
