# Supplementary material for: Prognostic value of GLIM-defined malnutrition in combination with hand-grip strength or gait speed for the prediction of postoperative outcomes in gastric cancer patients with cachexia
Source: BMC Cancer. 2024 Feb 23;24:253. doi: 10.1186/s12885-024-11880-z (PMC10885679; doi:10.1186/s12885-024-11880-z)
Supplement: Supplementary file 3 — Supplementary Material 3 [file 12885_2024_11880_MOESM3_ESM.docx]

**Supplementary Table 2. Influence of GLIM-defined malnutrition on cachexia patients categorized by severity of sarcopenia**

|  | Non-sarcopenia (n=282) | | | Sarcopenia, not severe (n=16) | | | Severe sarcopenia (n=58) | | |
| --- | --- | --- | --- | --- | --- | --- | --- | --- | --- |
| **Shor-term outcomes** | Non malnutrition (n =79) | GLIM-defined malnutrition (n =203) | P | Non malnutrition (n =2) | GLIM-defined malnutrition (n =14) | P | Non malnutrition (n =6) | GLIM-defined malnutrition (n =52) | P |
| **Total complications**^†^ | 16 (20.3%) | 50 (24.63%) | 0.436 | 1 (50%) | 5 (35.7%) | 1.000 | 4 (66.7%) | 26 (50%) | 0.732 |
| **Severe complications**^‡^ | 3 (3.8%) | 12 (5.9%) | 0.678 | 0 (0%) | 1 (7.1%) | 1.000 | 1 (16.7%) | 6 (11.5%) | 0.555 |
| **Length of postoperative stays, median (IQR), days** | 16.5 (18.25) | 16 (10) | 0.315 | 32.5 (-) | 15 (9) | 0.265 | 13 (5) | 13 (7) | 0.929 |
| **Costs, median (IQR), RMB** | 78208.67 (71772.61) | 75721.12 (28060.76) | 0.178 | 67861.54 (-) | 65748.20 (36852.31) | 0.634 | 58982.04 (19969.29) | 62466.09 (25615.90) | 0.251 |

IQR, interquartile range

The values in the table were number of patients and percent unless indicated otherwise.

^†^ Complications classified as grade II and above.

^‡^ Complications classified as grade III and above.

^*^ Statistically significant compared with the opposite group.
