# Supplementary material for: Prognostic value of GLIM-defined malnutrition in combination with hand-grip strength or gait speed for the prediction of postoperative outcomes in gastric cancer patients with cachexia
Source: BMC Cancer. 2024 Feb 23;24:253. doi: 10.1186/s12885-024-11880-z (PMC10885679; doi:10.1186/s12885-024-11880-z)
Supplement: Supplementary file 5 — Supplementary Material 5 [file 12885_2024_11880_MOESM5_ESM.docx]

**Supplementary Figure 1.** Survival curves of overall survival (a) and disease-free survival (b) in patients with moderate or severe malnutrition.

**Supplementary Figure 2.** Survival curves of overall survival and disease-free survival in patients with and without GLIM-defined malnutrition in the ‘non-sarcopenia’ (a, b), ‘sarcopenia, not severe’ (c, d), and ‘severe sarcopenia’ (e, f) sub-groups.
